# Post‐Exercise Circulatory Occlusion and the Human Muscle Metaboreflex: Principles, Pitfalls and Proxies

**DOI:** 10.1111/apha.70356

**Published:** 2026-11-09

**Authors:** Jeann L. Sabino‐Carvalho, Igor A. Fernandes, Paul J. Fadel, Lauro C. Vianna

**Affiliations:** ^1^ NeuroV̇ASQ̇—Integrative Physiology Laboratory, Faculty of Physical Education University of Brasília Brasília Brazil; ^2^ Human Neurovascular Control Laboratory, Department of Health and Kinesiology Purdue University West Lafayette Indiana USA; ^3^ Department of Kinesiology University of Texas at Arlington Arlington Texas USA; ^4^ Physical (In)activity and Health National Institute of Science and Technology‐INCT and CNPq Brasília Brazil

The human muscle metaboreflex contributes importantly to cardiovascular regulation during exercise, but its assessment remains indirect and highly dependent on experimental design. Here, we revisit classic experiments that established the physiological basis of post‐exercise circulatory occlusion (PECO), discuss how different methods interrogate distinct components of the reflex pathway, and highlight key principles that should guide the design and interpretation of human metaboreflex studies. Particular attention is given to the need for sufficient reflex activation, careful cuff placement, the amount of tissue rendered ischaemic, and the haemodynamic and mechanical consequences of vascular occlusion. We also consider alternative approaches, including manipulation of muscle blood flow during exercise, and discuss the distinction between physiological gain and ratios derived from proxy measures such as peripheral fatigue. Together, these examples illustrate that stronger inference regarding metaboreflex control depends on combining complementary approaches, appropriate control conditions, and careful physiological interpretation.

Metabolic changes within contracting skeletal muscle activate predominantly Group IV afferents, while some Group III afferents exhibit polymodal sensitivity or may be sensitized by the local metabolic milieu. This afferent input engages central autonomic circuits that increase sympathetic outflow, heart rate (HR), and arterial blood pressure (BP) [1, 2]. Characterizing the muscle metaboreflex can be challenging because its assessment relies on experimental manipulations and indirect measures of reflex activation. Much of our understanding derives from post‐exercise circulatory occlusion (PECO), in which supra‐systolic cuff inflation proximal to the active muscle before the end of contraction limits metabolite washout during recovery. When contraction stops, contributions from central command and the mechanoreflex decline, while metabolically sensitive afferent activation is sustained [1, 2, 3]. Nevertheless, cardiovascular responses during PECO remain subject to other autonomic influences. Fisher et al. (2010) [4], for example, showed that cardiac parasympathetic reactivation can mask metaboreflex‐mediated cardiac sympathetic effects on HR. Thus, isolating the muscle metaboreflex response can be challenging, highlighting the complexity of the integrated physiological response. In this context, BP provides an integrated cardiovascular readout, whereas MSNA, when available, offers a more direct measure of sympathetic outflow to skeletal muscle.

Alam and Smirk first showed that arresting circulation to active muscle just before exercise ceased led to a sustained exercise‐induced pressor response, suggesting a reflex arising from exercising muscle [1]. Decades later, Mark et al. directly recorded postganglionic muscle sympathetic nerve activity (MSNA) and showed that during PECO, BP and MSNA remained elevated after HR had returned toward resting values (Figure 1A), whereas cuff inflation without preceding exercise did not reproduce this response [3]. Together, these findings provided foundational evidence that a metabolically sensitive reflex originating in skeletal muscle can evoke marked increases in MSNA and BP that persist during PECO.

**FIGURE 1:**
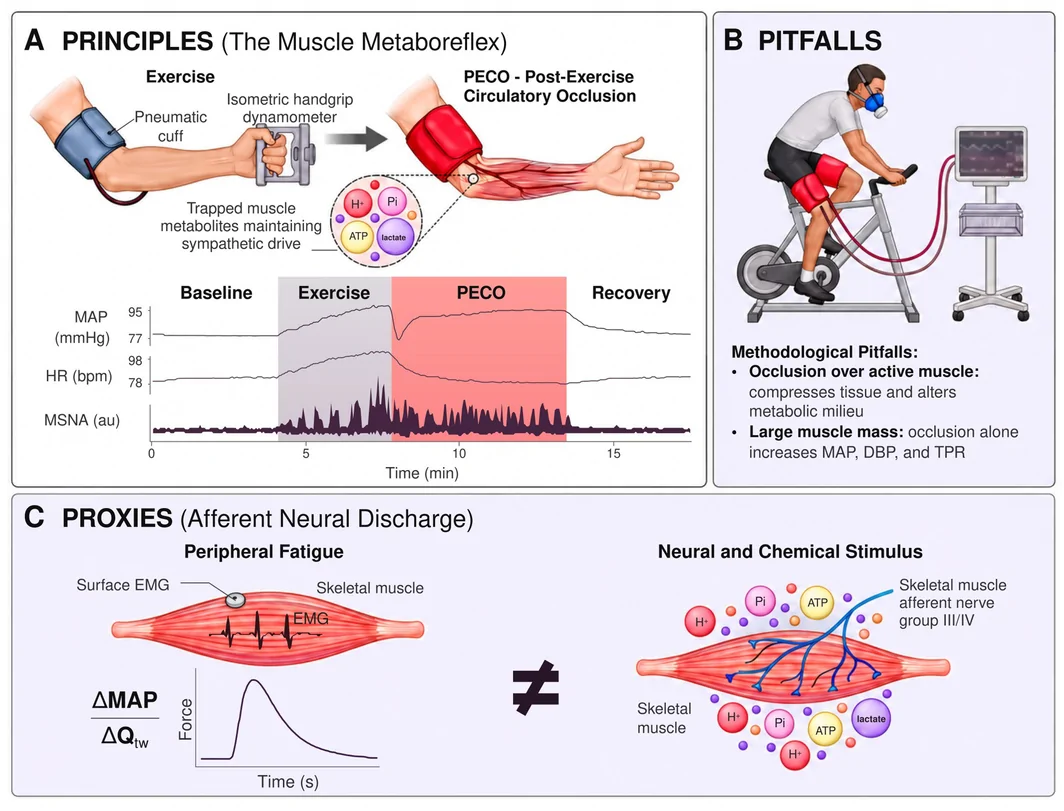
Human muscle metaboreflex assessment: Principles, pitfalls, and proxies. (A) Isometric handgrip followed by post‐exercise circulatory occlusion (PECO). Suprasystolic occlusion established before contraction ends limits metabolite washout from the previously active muscle. During PECO, mean arterial pressure (MAP) and muscle sympathetic nerve activity (MSNA) remain elevated as heart rate (HR) returns toward baseline. (B) Methodological pitfalls associated with cuff placement and the vascular territory occluded. A cuff positioned directly over the active musculature compresses tissue and may alter local metabolic conditions. Occlusion of large vascular territories can itself produce haemodynamic changes, complicating attribution of the pressor response to metaboreflex activation. (C) Peripheral fatigue and afferent stimulation represent distinct physiological processes. Loss of electrically evoked quadriceps twitch force (ΔQtw) indexes peripheral fatigue but does not directly quantify the chemical stimulus reaching Group III/IV afferent endings or their neural discharge. Accordingly, ΔMAP/ΔQtw expresses the pressor response relative to peripheral fatigue. ATP, adenosine triphosphate; DBP, diastolic blood pressure; EMG, electromyography; Pi, inorganic phosphate; TPR, total peripheral resistance.

Available methods interrogate distinct steps between muscle metabolism and cardiovascular responses. On the stimulus side, ^31^P magnetic resonance spectroscopy (^31P‐MRS) assesses intracellular metabolism, microdialysis samples selected components of the interstitial milieu, muscle blood flow characterizes or alters perfusion and metabolite washout, and evoked twitch force reflects peripheral fatigue. MSNA measures sympathetic outflow, whereas BP represents the integrated haemodynamic response. MacLean et al. combined microdialysis, MSNA, and haemodynamic measurements and showed different temporal patterns for interstitial lactate, phosphate, K^+^, and H^+^ during exercise and recovery [5], consistent with metaboreflex activation reflecting the combined effects of multiple metabolites. Complementary sympathoexcitatory maneuvers such as the cold pressor test provide a useful non‐exercise control for comparison. Preserved MSNA and BP responses to this test despite augmented or attenuated PECO responses argue against generalized differences in sympathetic responsiveness and support a more selective alteration within the muscle metaboreflex pathway.

A few practical principles can help address the inherent challenges of assessing the muscle metaboreflex in humans. The first principle is to use an exercise stimulus sufficient to robustly engage the metaboreflex. Fadel et al. compared static handgrip at 33% and 45% of maximal voluntary contraction (MVC). In healthy controls, peak MSNA and BP increased by approximately 171% and 24% at 33% MVC, and by 379% and 37% at 45% MVC, respectively [6]. Differences in exercise intensity and duration may therefore help explain discrepant findings across studies. At a given contraction duration, 30% MVC may fall below the metaboreflex activation threshold in some individuals, requiring a higher intensity. This is particularly relevant in patient comparisons, where obtaining a true MVC may be challenging, and underestimation yields a lower target force at the same prescribed relative percentage. Thus, a modest response may reflect insufficient reflex engagement rather than metaboreflex dysfunction.

A second principle is to minimize the maneuver's haemodynamic and mechanical effects, especially when larger tissue volumes are rendered ischaemic. Small‐muscle‐mass models can be advantageous for isolating neurally mediated metaboreflex responses because they minimize haemodynamic effects introduced by vascular occlusion. Whenever possible, cuffs should be placed proximally to, rather than directly over, the primary exercising muscle mass to minimize direct tissue compression. This is particularly relevant during knee‐extension or cycling, in which a thigh cuff may overlap substantially with active musculature, whereas calf exercise allows thigh occlusion without directly compressing the exercising muscles. Furthermore, bilateral leg occlusion can also alter vascular conductance, venous return, central blood volume, and baroreflex loading. Rowell et al. showed that resting leg occlusion could increase BP without changes in HR, ventilation, or forearm blood flow [7]. During cycling, Nóbrega et al. found that bilateral thigh cuff inflation reduced end‐diastolic volume and blunted the rise in stroke volume [8]. Lis et al. showed that bilateral thigh occlusion alone increased MAP, DBP, and total peripheral resistance; notably, the increase in total peripheral resistance was similar to that observed when occlusion was superimposed on passive cycling [9]. Large‐muscle‐mass models may therefore offer greater ecological validity but require caution when separating neurally mediated metaboreflex responses from haemodynamic and mechanical effects of occlusion itself (Figure 1B).

Although much of the human literature has relied on PECO, the metaboreflex can also be studied by manipulating muscle perfusion during ongoing exercise. Based on studies by O'Leary in dogs, Ichinose et al. [10] progressively reduced and clamped forearm blood flow during rhythmic handgrip. Initial reductions produced little cardiovascular response, but below an identifiable threshold, further reductions elicited graded increases in BP and HR and reductions in total vascular conductance. Reflex gain was estimated from the slope of this response, illustrating that physiological gain is most meaningfully inferred when an experimentally controlled input is related to the resulting output over a defined response range.

This distinction is relevant to a recent study of Kouzkouz et al., who normalized the arterial pressure response during PECO to the reduction in electrically evoked quadriceps twitch force (ΔQtw), defining ΔMAP/ΔQtw as “metaboreflex gain” [11]. Their approach addresses an important problem because the same relative exercise intensity does not necessarily generate the same intramuscular perturbation across individuals. However, twitch‐force loss is a proxy for peripheral fatigue, not a calibrated measure of the chemical stimulus reaching Group III/IV afferent endings or of afferent neural discharge. ΔMAP/ΔQtw therefore represents pressure relative to peripheral fatigue rather than a direct measure of metaboreflex gain (Figure 1C). Importantly, before normalization, the overall PECO responses showed no significant sex or interaction effect for ΔMAP, cardiac output, total peripheral resistance, or stroke volume; only HR differed between males and females. Nevertheless, the authors emphasize greater pressor responses in males at selected levels of metaboreflex activation and subsequently frame these findings as evidence of a sex difference that was abolished by normalization. This selective emphasis has important interpretative consequences because it gives greater weight to isolated comparisons than to the overall statistical pattern. Accordingly, the disappearance of those selected differences after normalization should not, by itself, be viewed as evidence that ΔMAP/ΔQtw represents physiological metaboreflex gain.

Fully characterizing the human muscle metaboreflex is challenging, but carefully controlled experiments allow meaningful assessment. Stronger inference requires applying physiological principles, minimizing experimental confounds, and interpreting proxies cautiously. Complementary maneuvers such as the cold pressor test can help determine whether altered PECO responses reflect metaboreflex‐specific changes or more generalized differences in sympathetic or vascular responsiveness. Progress may therefore depend more on combining complementary measurements and controlled physiological manipulations than on trying to identify a universal “metaboreflex index”.

## Author Contributions

**Jeann L. Sabino‐Carvalho:** conceptualization, writing – original draft, writing – review and editing, project administration. **Igor A. Fernandes:** writing – review and editing. **Paul J. Fadel:** writing – review and editing. **Lauro C. Vianna:** conceptualization, writing – review and editing.

## Funding

J.L.S.‐C. receives research support from the National Council for Scientific and Technological Development (CNPq; grant nos. 315309/2025‐3 and 446406/2024‐4). I.A.F. was supported by the Arthur C. Guyton Award for Excellence in Integrative Physiology from the American Physiological Society and by the American Heart Association (grant no. 24CDA1273371). P.J.F. was supported by the Moritz Chair in Geriatrics, the College of Nursing and Health Innovation, the University of Texas at Arlington. L.C.V. receives research support from the CNPq (grant no. 307764/2022‐2), from the National Institute of Science and Technology‐INCT, Physical (In) activity and Health (CNPq grant no. 408899/2024‐7), and from the Federal District Research Support Foundation (FAPDF; grant no. 00193‐00001619/2024‐61).

## Conflicts of Interest

The authors declare no conflicts of interest.

## Data Availability

Data sharing not applicable to this article as no datasets were generated or analysed during the current study.
